# Bullous Cutaneous Larva Migrans of the Foot

**DOI:** 10.4269/ajtmh.23-0750

**Published:** 2024-02-27

**Authors:** Ashton D. Hall, Keith M. Luckett, Kelli M. Williams

**Affiliations:** Division of Infectious Diseases, Department of Internal Medicine, University of Cincinnati College of Medicine, Cincinnati, Ohio

**Figure 1.**
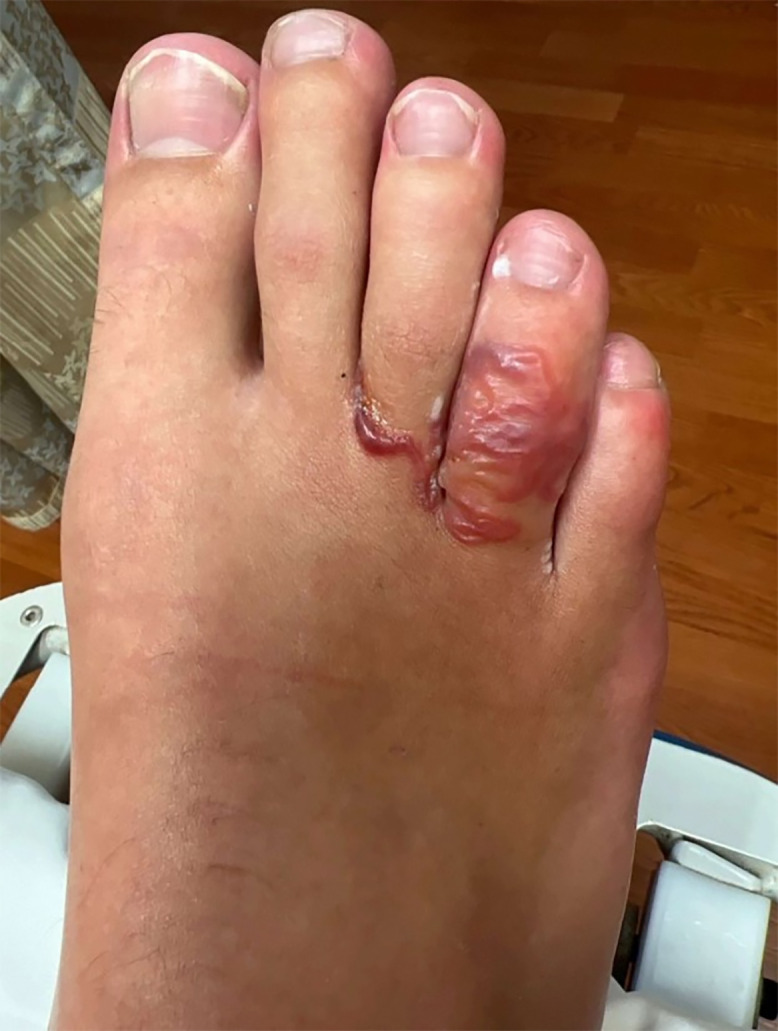
Erythema and blistering of the third and fourth toes on initial presentation.

**Figure 2.**
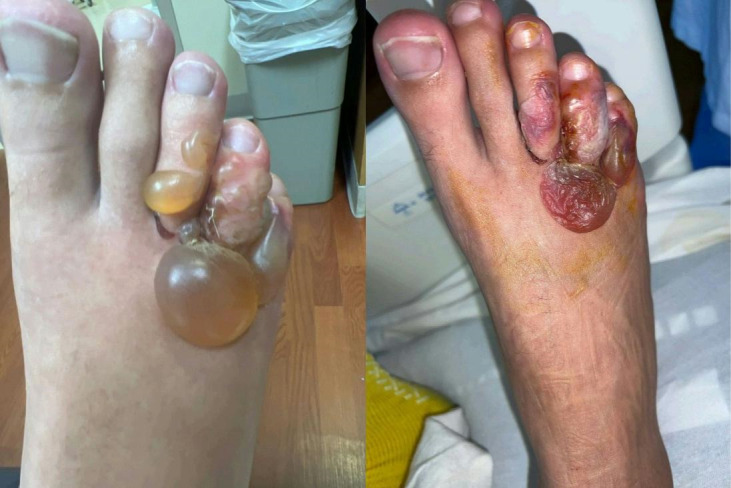
Progressively worsening edema and blistering of the lesser toes despite antibiotic treatment. Picture was taken before and after aspiration of the bullae.

**Figure 3.**
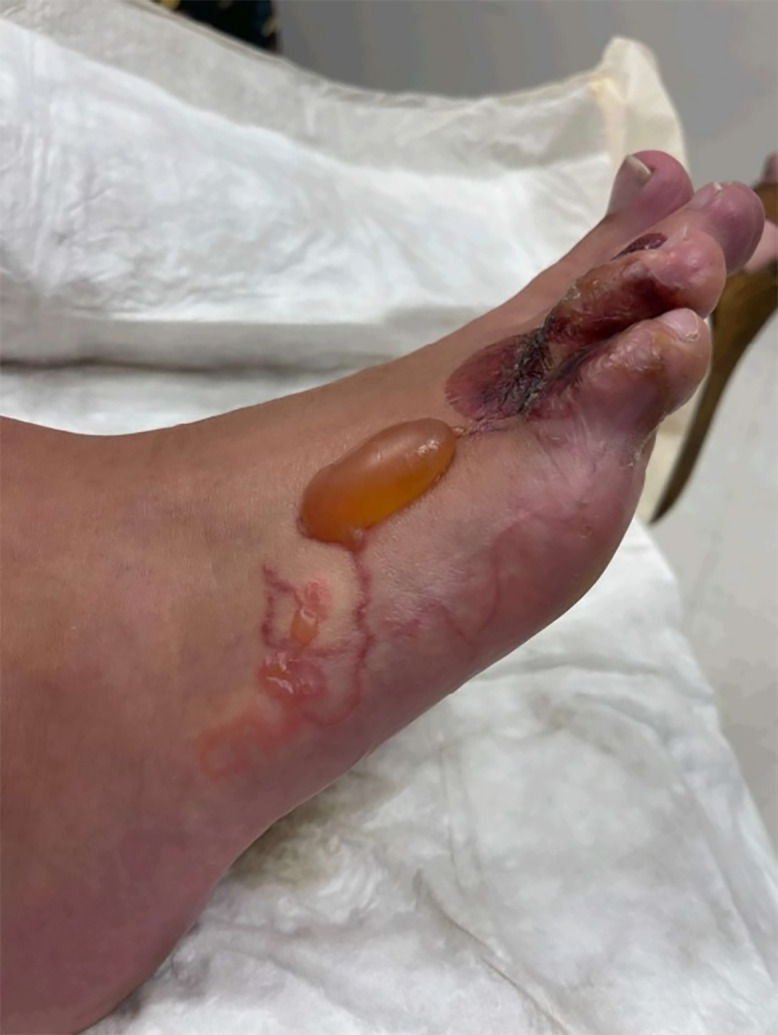
Lateral view of the foot showing red serpiginous streaks from the lesser toes to the lateral midfoot and a dorsal serous blister, resulting in the diagnosis of cutaneous larva migrans.

**Figure 4.**
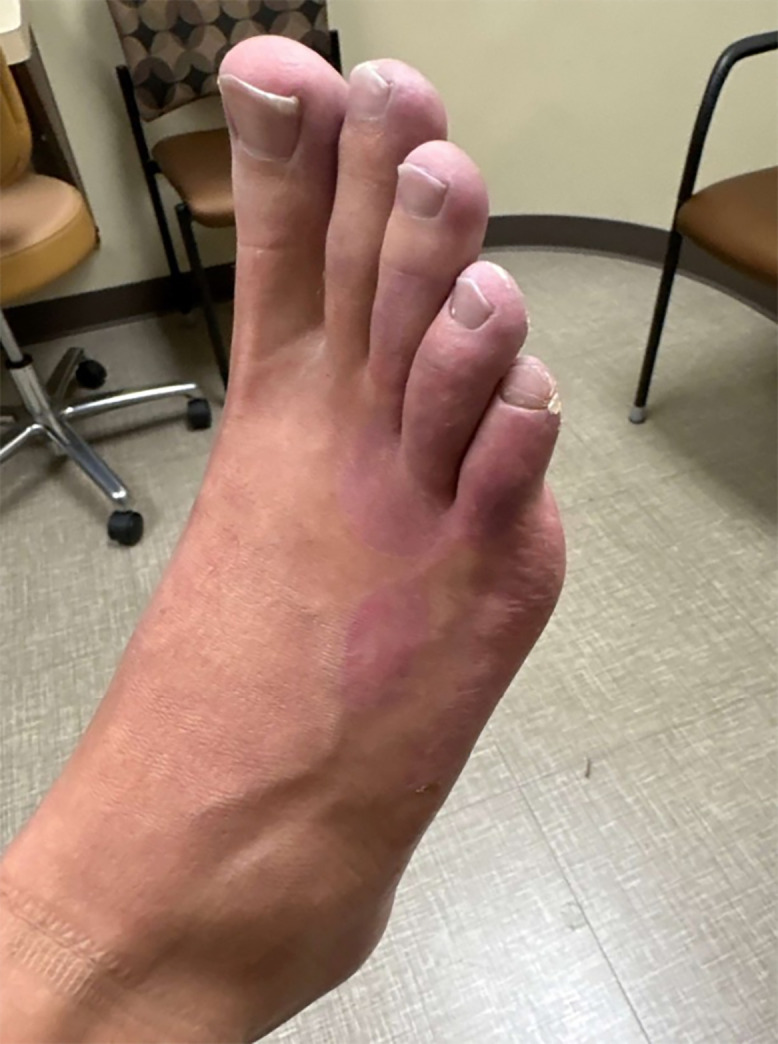
Resolution of bullae and serpiginous tracts following ivermectin. Picture was taken five weeks after the patient’s initial presentation.

A 25-year-old man presented with a painful and pruritic rash on the right foot that had been present for several weeks. Examination revealed erythema and blistering of the toes without systemic symptoms (Figure 1). Travel history included a recent vacation to Jamaica, where the patient walked barefoot on the beach. He also reported a trip to Red River Gorge in eastern Kentucky, where he went hiking but denied getting his feet wet. He denied traumatic injury. An over-the-counter antifungal cream did not provide relief. On his initial presentation to the emergency department, he was diagnosed with atypical cellulitis, prescribed cephalexin and trimethoprim-sulfamethoxazole, and discharged. The patient returned 1 week later with worsening bullous lesions of the right foot, tenderness to palpation, and decreased capillary refill (Figure 2). Because of concern for *Vibrio* or *Aeromonas* soft-tissue infection, the patient was admitted and prescribed doxycycline and cefpodoxime. Radiographs showed a well-defined soft-tissue density projecting along the dorsal foot near the third and fourth metatarsophalangeal joint. Acid fast, anaerobic, and fungal cultures were negative after aspiration of the bullae. Magnetic resonance imaging ruled out osteomyelitis, and the patient was discharged on hospital day 3. At outpatient follow-up 1 week later, the patient’s rash had progressed to a red serpiginous lesion (Figure 3). Given epidemiologic risk factors and clinical history, the patient was diagnosed with cutaneous larva migrans (CLM) and treated with 200 mcg/kg of ivermectin for 2 days. His blistering lesions and pruritus resolved within 3 weeks (Figure 4).

Cutaneous larva migrans, or creeping eruption, is one of the most common, albeit underrecognized, skin conditions reported in travelers returning from tropical and subtropical countries.^1^ This infection results from the penetration and migration of filariform larvae through the epidermis.^1^ Typical manifestations are intensely pruritic, slightly raised serpiginous rashes on the distal extremities, reflecting the path of the parasite.^2^ Bullae are atypical for CLM, accounting for only 3% of cases.^2^^–^^4^ Mechanisms responsible for bullae formation include the release of histolytic larval enzymes or a delayed hypersensitivity reaction to unknown larval antigens.^2^ Diagnosis is predominantly clinical and based on the patient’s travel to endemic regions, exposure to contaminated soil or sand, and a raised erythematous tract.^1^ Larva currens is the primary alternative diagnosis; however, it elicits severe urticaria owing to rapid larval migration at 5–15 cm per hour compared with 1–2 cm per day for CLM.^5^ First-line treatment includes oral albendazole or ivermectin, whereas antihistamines and corticosteroids may provide symptomatic relief.^6^
